# Our New York Letter

**Published:** 1887-09

**Authors:** 


### Correspondence.

### OUR NEW YORK LETTER.

A NEW YORK PHYSICIAN ARRESTED FOR PRACTICING DENTISTRY—THE COURTS SUSTAIN HIM—A NEW YORK DOCTOR GETS PERMISSION FROM THE MAYOR TO PRACTICE PASTEUR'S METHOD ON DOGS IN THE CITY POUND, BUT MR. HENRY BERGH SAYS NO—INCREASE OF TYPHOID FEVER IN NEW YORK—THE NEW PRESIDENT OF THE BOARD OF HEALTH AFTER THE DRUG CLERKS—OTHER REFORMS, ETC.

*To the Editors of the Atlanta Medical and Surgical Journal:*

The question as to whether doctors of medicine may lawfully practice dentistry was brought up some days ago by Dentist F. W. White, who had Dr. Orlando Bradford, a graduate of the Eclectic Medical College, arrested and brought into court on the charge of practicing dentistry without a diploma from the State of New York Dental Medical Society. This, it was claimed, rendered him guilty of a violation of the law under the registration act, which was passed June 20, 1879. The defence was that the physician by holding a diploma from a reputable medical college was therefore entitled to practice dentistry. The prosecution then showed a list of forty-two reputable dental and medical colleges which the Dental Society recognized, but the Eclectic College was not on the list. Judge Gorman remarked that the College of Physicians and Surgeons was also omitted from the list. The case was then adjourned to enable the justice to obtain more particular information relating to this subject. When the adjourned hearing came up, Judge Gorman said he had been under the impression that the dentist's lawyer was acting for the New York State Dental Society in making this complaint against Dr. Bradford, but he had recently received a letter from the censor of this society denying that this organization had any connection with the case. While the lawyer was trying to explain that he had not meant to deceive anybody, he was interrupted by his honor, who ordered that Dr. Bradford be discharged, and added that, in his opinion, a regular graduated physician from a reputable medical college had a perfect right to practice dentistry if he wished to do so.

Another physician of this city is anxious to try the Pasteur method. Dr. Leo Sommer, who graduated from the Medical University of Buda-Pesth, Hungary, has been trying to get the Mayor's permission to make use of a part of the dog pound for the purpose of carrying on experiments in inoculation as a preventive of hydrophobia. The Mayor, after consulting with the President of the Board of Health, gave the necessary permission, and the doctor expected to commence work immediately. The receipt of a letter from Mr. Henry Bergh threw a wet blanket on the doctor's hopes. This Mr. Bergh is President of our Society for the Prevention of Cruelty to Animals, and in this capacity he never fails to attempt rescuing all dumb beasts from whatever has the appearance of cruelty. In this instance he does not think that the results obtained in similar lines of research warrant any such wholesale infliction of suffering. Dr. Sommer has been experimenting for a period of three years by inoculating dogs as a preventive against hydrophobia. His theory is that if all dogs could be inoculated, it would be entirely unnecessary for any patients ever to place themselves under Pasteur's method of treatment. The dog to be inoculated is muzzled and placed in an apparatus which keeps him on his back and holds the legs fast. An abrasion is made with a lance between the animals forelegs and some of the virus applied in this situation. The effects of this inoculation appear quite rapidly and resemble closely the symptoms of hydrophobia, but run a short course and end in recovery. After the animal has had this attack he is proof against rabies, according to Dr. Sommer. Originally the doctor obtained this virus from a rabid dog, but since then his supply has been taken from inoculated animals, and even this he claims is too powerful and requires dilution. The law on this subject, which has suddenly interfered with these experiments, is that no such investigations can be made except under the authority of the faculty of some regularly incorporated medical college of the State of New York.

An unusually large number of cases of typhoid fever have occurred here during the few days which have just passed. The cause of this increase has not been determined, but various theories have been advanced. As many of our streets have been dug up by the Steam Heating Company, and for the purpose of burying the telegraph wires, during the excessive heat and humidity of the present summer, this state of affairs is considered to a certain degree responsible for these cases of sickness. Some doctors are inclined to believe that the excessive use of ice water, together with tenement house effluvia, should also shoulder part of the responsibilty.

Since the appointment of Mr. Bayles to the presidency of the Board of Health many important reforms have taken place in this department. What is known as the drug danger will receive due consideration. It appears that at present there are about five hundred persons engaged in dispensing drugs who have never graduated in pharmacy. As all such persons hold responsible positions and should be thoroughly competent, they will hereafter be obliged to pass an examination and register according to the law. Only those who have owned stores and been in the business a considerable time will be exempt from such examination, for their years of service will be taken into consideration, but they will nevertheless be required to register. For some time now drugs and patent medicines have been largely sold in many of our most prominent fancy stores by boys and girls who are entirely inexperienced in this branch of trade, and the above law will also include these cases and end this promiscuous selling of medicines. An effort will also be made to prevent public funerals taking place in cases of death from contagious diseases.

The law on this subject has seldom been enforced, but only recently an undertaker and the father of a child who died with diphtheria were arrested for violating the sanitary code by permitting a public funeral. Ignorance of the law will not be accepted as an excuse, and hereafter all death certificates will contain the rules on this subject, and when deaths from contagious diseases are reported a copy of the law will be sent to the house where such death occurred. All physicians employed as sanitary inspectors in the health department will be required to devote the hours between 9. A. M. and 4 P. M. to the city's service, and relinqush all private practice during these hours. Many of these physicians have fair practices, but they formerly have been able to attend to the business of the health board and look after private patients in addition. Now they are practically asked to set aside private practice, and are only given a salary of $1,200 a year. As a result many have sent in their resignations, and it is expected that more will follow. It is the determination of President Bayles to enforce the new rules, and detectives have been engaged in watching the inspectors, several of whom have been brought up with rather a sharp turn. A. F.

New York, August, 1887.

